# Ultrasound-guided quadratus lumborum block for postoperative analgesia in a pediatric patient with congenital hip dislocation: a case report

**DOI:** 10.3389/fped.2026.1830713

**Published:** 2026-05-19

**Authors:** Man Li, Fu Yao, Zhijun Qin

**Affiliations:** 1 Department of Anesthesiology, Sichuan Orthopedic Hospital, Chengdu, Sichuan, China; 2 Department of Orthopedics, Sichuan Orthopedic Hospital, Chengdu, Sichuan, China

**Keywords:** case report, developmental dysplasia of the hip, pediatric anesthesia, perioperative management, ultrasound-guided quadratus lumborum block

## Abstract

Developmental dysplasia of the hip (DDH) is a common congenital pediatric orthopedic malformation that often requires open reduction combined with pelvic osteotomy, resulting in severe postoperative pain. Pediatric patients under 2 years old have immature anatomical and physiological characteristics, high sensitivity to opioid analgesics, and an inability to subjectively express pain, which makes perioperative analgesia and anesthesia management a major clinical challenge. Traditional opioid-based analgesic regimens carry a high risk of adverse reactions such as respiratory depression and restlessness, which hinder postoperative recovery. This case reports the optimized perioperative anesthesia and analgesia management strategy for a 19-month-old female infant (12 kg, ASA Ⅰ) with right DDH undergoing open hip reduction and pelvic osteotomy, and preliminarily explores the clinical application value, efficacy and safety of ultrasound-guided type Ⅱ QLB in infant DDH surgery. A composite anesthesia strategy of general anesthesia combined with ultrasound-guided type Ⅱ QLB (0.2% ropivacaine, 0.5 mL/kg, total 6 mL) was adopted, with optimized perioperative management including precise airway selection, targeted vital sign monitoring, and multimodal postoperative analgesia (PCIA+on-demand oral acetaminophen). Pain was evaluated using the FLACC behavioral scale, and vital signs and adverse reactions were closely monitored postoperatively. The infant's 24-hour postoperative FLACC scores were all ≤2 points without the need for rescue analgesics, with significantly reduced opioid consumption (intraoperative sufentanil 6 *μ*g, 24-hour PCIA tramadol 70 mg) compared with traditional regimens. Vital signs remained stable postoperatively, with no adverse reactions such as restlessness, nausea/vomiting, urinary retention, or local hematoma. The infant achieved independent turning and sitting at 48 h postoperatively and was discharged uneventfully at 14 days with no long-term complications, and parental analgesia satisfaction was 9/10. Ultrasound-guided type Ⅱ QLB combined with general anesthesia is a safe and effective perioperative management strategy for infant DDH surgery, which addresses pediatric-specific perioperative analgesia challenges, reduces opioid-related adverse reactions, and improves postoperative comfort. Strict control of local anesthetic dosage, proficient ultrasound localization for immature pediatric anatomy, and multimodal analgesia are the key to successful perioperative management, which provides a valuable clinical reference for pediatric orthopedic anesthesia practice.

## 1 Introduction

Developmental dysplasia of the hip (DDH, formerly congenital hip dislocation) is the most common congenital malformation of the pediatric hip joint, with open reduction combined with pelvic osteotomy as the first-line surgical treatment for severe cases (1). This surgery involves extensive soft tissue dissection and pelvic bone osteotomy, leading to severe somatic pain that can trigger restlessness, delayed wound healing, and an increased risk of chronic pain in pediatric patients (2). Perioperative anesthesia and analgesia management for infant DDH surgery (≤2 years old) presents unique pediatric-specific challenges, including anatomical immaturity (underdeveloped lumbar paraspinal muscles, narrow airway, and soft tracheal cartilage), physiological sensitivity to opioids (high opioid receptor density and immature respiratory center), limited pain assessment (non-verbal infants unable to report pain subjectively), and poor perioperative compliance. Traditional analgesic regimens relying on systemic opioids fail to address these challenges and are associated with a high incidence of adverse reactions.

Quadratus lumborum block (QLB) is a novel regional nerve block technique that provides extensive analgesia for the hip and lower extremity by blocking the lumbar plexus and thoracolumbar sympathetic nerves, with the advantages of minimal motor block and wide analgesic coverage (1). A recent meta-analysis of 16 RCTs involving 1,061 pediatric patients further verified that QLB significantly reduced the need for rescue analgesia at 12 and 24 h postoperatively, and Type Ⅱ QLB uniquely prolonged the time to the first rescue analgesia, which is the most suitable QLB subtype for pediatric hip surgery (3).Ultrasound guidance enables clear identification of immature pediatric anatomical structures, reducing the risk of block-related complications and making it the preferred approach for pediatric regional anesthesia (4). Existing clinical studies on QLB for pediatric DDH surgery mostly focus on school-age children or the application of type Ⅲ/Ⅳ QLB, while there is a lack of detailed, standardized reports on the full perioperative management of type Ⅱ QLB in infants under 2 years old. This population has the most immature anatomical and physiological development, the highest risk of opioid-related adverse reactions, and the most difficult pain assessment and anesthesia management, which is the key and difficult point of clinical pediatric orthopedic anesthesia. This case helps address the aforementioned research gap by reporting a complete, infant-tailored anesthesia and analgesia strategy for 19-month-old DDH surgery, including detailed operational specifications, drug dosage parameters, and full-process safety control points, thereby providing a practical clinical reference for pediatric anesthesiologists.

This case reports the optimized perioperative anesthesia and analgesia management for a 19-month-old infant with DDH undergoing open hip surgery, focusing on infant-specific airway management, ultrasound-guided QLB operation, and multimodal analgesia. We discuss the clinical rationale and key points of this management strategy to provide evidence-based perioperative management references for infants with DDH.

## 2 Case presentation

### 2.1 Preoperative evaluation and preparation (pediatric-specific optimization)

A 19-month-old female infant, weighing 12 kg and measuring 84 cm in height, with ASA physical status Ⅰ, was admitted with a 1-year history of right hip dislocation and a confirmed diagnosis of right DDH. The infant had an unremarkable medical history, no history of neuromuscular disease, coagulation dysfunction, or drug allergy. Preoperative laboratory tests (complete blood count, coagulation function, liver and kidney function) and electrocardiography were all normal. Orthopedic assessment showed limited right hip movement, no local skin infection, and good mental status with general compliance.No diagnostic challenges were encountered.

For this infant, the surgical procedure involved extensive soft tissue dissection around the hip joint and pelvic osteotomy, which will cause severe moderate to severe postoperative pain. Considering the infant's age (19 months, <2 years old), immature respiratory center, high sensitivity to opioids, and inability to subjectively express pain, we selected ultrasound-guided type Ⅱ QLB as the core of the perioperative analgesia strategy, and the rationale for choosing this technique over alternative regional block methods is as follows:

1. Compared with caudal block: Traditional caudal block is commonly used in pediatric lower extremity surgery, but it has a 20%∼30% incidence of urinary retention in pediatric patients (1), which will affect the postoperative nursing of infants and increase the risk of urinary tract infection; meanwhile, caudal block cannot fully cover the sensory innervation of the hip joint and pelvic osteotomy area, with limited analgesic efficacy for DDH open surgery. A 2025 meta-analysis further confirmed that QLB significantly reduced the 24-hour postoperative rescue analgesia rate compared with caudal block in pediatric abdominal and hip surgery, with no significant difference in the incidence of complications between the two techniques (5).
2. Compared with supra inguinal fascia iliaca block: This block can cover the anterior branch of the lumbar plexus, but it has incomplete block of the posterior branch of the lumbar plexus and thoracolumbar sympathetic nerves, with insufficient analgesic coverage for the posterior hip and pelvic osteotomy area, and the analgesic duration is shorter than QLB (2).
3. Compared with pericapsular nerve group (PENG) block: PENG block can provide targeted analgesia for the anterior hip capsule, but its analgesic coverage is limited, and it cannot cover the pelvic osteotomy area and lateral thigh surgical incision of DDH open surgery; meanwhile, the anatomical structure around the hip joint of infants is narrow, and the puncture risk of PENG block is higher than that of QLB (6).

Type Ⅱ QLB could simultaneously block the lumbar plexus (L1∼L4) and thoracolumbar sympathetic nerves, with wide analgesic coverage highly matched with the surgical area of DDH open surgery, minimal motor block, and low incidence of urinary retention, which is the optimal regional block choice for this infant. The uniqueness of this case is that the whole perioperative management strategy is completely tailored to the anatomical and physiological characteristics of infants under 2 years old, including precise airway management, ultrasound block operation parameters adapted to infant anatomy, low-dose opioid-sparing analgesic regimen, and targeted pediatric safety monitoring, to maximize the balance of analgesic efficacy and safety.

Pediatric-specific preoperative preparation was implemented in accordance with pediatric anesthesia guidelines: (1) Strict fasting and water deprivation (6 h for solid food, 2 h for clear liquids) to reduce the risk of perioperative aspiration, a major fatal complication in infants; (2) Intravenous midazolam 0.5 mg (0.04 mg/kg) for mild preoperative sedation to improve cooperation, avoiding excessive sedation that could suppress respiration; (3) Establishment of peripheral intravenous access with a 24G indwelling needle, suitable for the infant’s vessel diameter, to ensure unobstructed intraoperative drug administration and fluid replacement.

### 2.2 Anesthesia induction and airway management (infant airway specificity)

Upon entering the operating room, standard pediatric anesthesia monitoring was initiated: electrocardiography, non-invasive blood pressure (BP), pulse oxygen saturation (SpO₂), and end-tidal carbon dioxide partial pressure (PETCO₂) (mandatory for early detection of respiratory depression in pediatric patients).

Anesthesia induction dosages were tailored to the infant's actual body weight (the gold standard for pediatric drug dosing): intravenous propofol 30 mg (2.5 mg/kg), sufentanil 4 μg (0.3 μg/kg), and rocuronium 10 mg (0.9 mg/kg). After mask positive pressure oxygenation, orotracheal intubation was performed with an uncuffed 3.5 mm internal diameter endotracheal tube (suitable for infants <2 years old with immature tracheal cartilage) at a depth of 13 cm (tip at the mid-trachea). Mechanical ventilation was initiated with infant-specific parameters: tidal volume 8 mL/kg, respiratory rate 20 bpm, inspiratory-expiratory ratio 1:2, maintaining PETCO₂ at 35∼45 mmHg (the normal range for infants) to avoid hypercapnia or hypocapnia.

### 2.3 Ultrasound-guided type Ⅱ QLB (pediatric anatomical adaptation)

**Figure 1:**
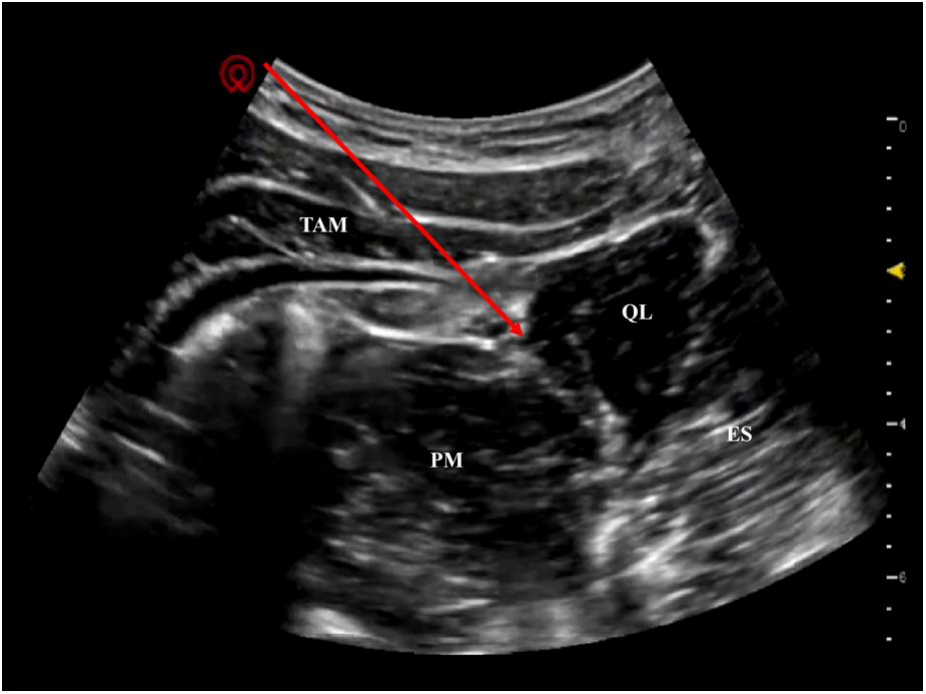
Ultrasound imaging of the target fascial space for type Ⅱ QLB in a 19-month-old infant. QL, quadratus lumborum; ES, erector spinae; TAM, transversus abdominis muscle; PM, psoas major muscle (red arrow indicates the target fascial space for type Ⅱ QLB).

The block was performed after successful tracheal intubation and stable general anesthesia, with the infant in the left lateral decubitus position (right waist exposed) and routine skin disinfection and sterile draping. A high-frequency linear ultrasound probe (6∼13 MHz, optimal for superficial pediatric soft tissue imaging) was placed at the intersection of the right midaxillary line and the subcostal margin. Real-time ultrasound clearly identified key anatomical structures adapted for the infant's thin soft tissue: quadratus lumborum (fusiform hypoechoic structure), erector spinae muscle, transvs. abdominis muscle, and psoas major muscle, and confirmed the fascial space between the posterior edge of the quadratus lumborum and erector spinae muscle (the target for type Ⅱ QLB) (Figure 1).

**Figure 2:**
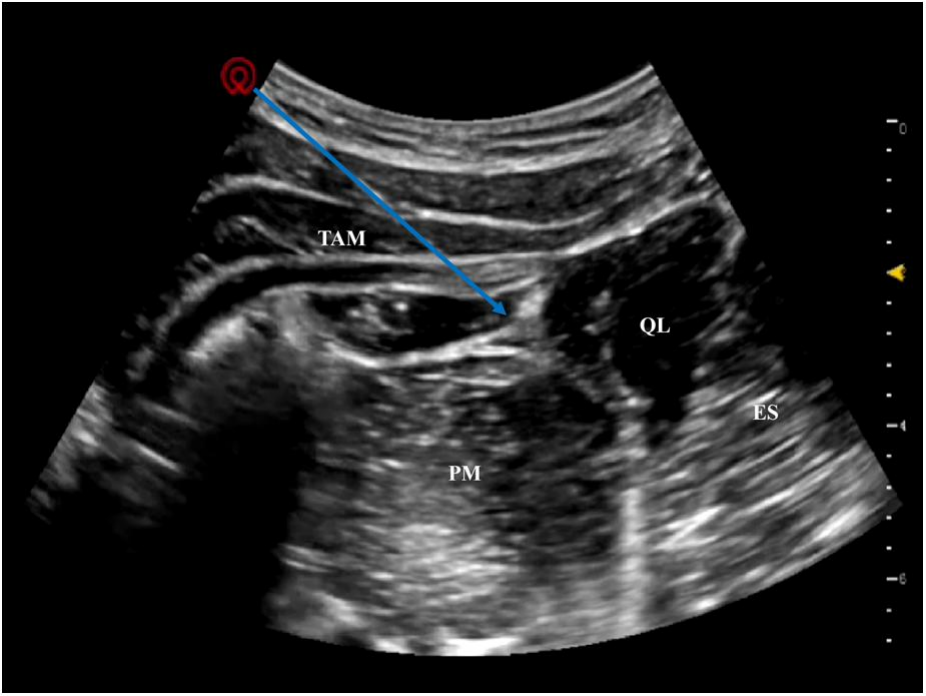
Ultrasound imaging of satisfactory 0.2% ropivacaine diffusion in the type Ⅱ QLB target space. Blue arrow indicates the diffusion range of the local anesthetic in the target fascial space, with no spread to adjacent blood vessels or abdominal organs.

An in-plane puncture technique was used with a 22G nerve block needle (thin needle to reduce local tissue damage). The needle tip was advanced to break through the posterior fascia of the quadratus lumborum and enter the target space. After negative aspiration (no blood or cerebrospinal fluid to rule out intravascular or intrathecal injection), 6 mL of 0.2% ropivacaine (0.5 mL/kg) was slowly injected (4). Ultrasound confirmed satisfactory diffusion of the local anesthetic in the target space with no spread to blood vessels or abdominal organs. Ten minutes after injection, gentle tactile stimulation of the right hip and lateral thigh skin showed no limb withdrawal response, confirming an effective sensory block before surgery initiation (Figure 2).

### 2.4 Intraoperative anesthesia maintenance and monitoring (pediatric vital sign targeting)

Anesthesia was maintained with inhaled sevoflurane (alveolar concentration 0.8%∼1.0%) combined with a continuous intravenous propofol infusion (4 mg·kg⁻^1^·h⁻^1^), avoiding high-concentration inhaled anesthetics to reduce neurotoxicity in the developing infant brain. Intraoperative additional sufentanil 2 μg (total 6 μg) was administered only for surgical stimulation to minimize opioid use.

Intraoperative vital signs were maintained within the infant's normal range: heart rate (HR) 100∼120 bpm, BP 85∼100/50∼65 mmHg, PETCO₂ 35∼45 mmHg. Intraoperative blood loss was approximately 100 mL, with homologous blood transfusion of 100 mL (volume replacement based on the infant's blood volume, ∼80 mL/kg) to maintain hemodynamic stability.

### 2.5 Surgical and postoperative perioperative management

The surgery lasted 180 min with stable intraoperative vital signs and no additional opioid analgesics administered. Patient-controlled intravenous analgesia (PCIA) was connected 30 min before the end of surgery with an infant-tailored formula: tramadol 200 mg + normal saline 100 mL, with a background dose of 1 mL/h, single bolus of 0.5 mL, and lock time of 15 min (a low background dose to avoid excessive tramadol accumulation).

After spontaneous respiration recovery and consciousness arousal, the endotracheal tube was extubated, and the infant was transferred to the post-anesthesia care unit for 30 min of observation. Postoperative monitoring included continuous HR, BP, and SpO₂, with regular FLACC pain score assessments (every 4 h within 24 h) and Ramsay sedation score assessments. Multimodal analgesia was implemented: continuous PCIA infusion combined with oral acetaminophen 15 mg/kg (on demand, repeat every 6 h). Rescue analgesia was set for a FLACC score ≥4 points. Postoperative adverse reaction monitoring focused on pediatric QLB and opioid-related complications: restlessness, nausea/vomiting, urinary retention, motor block, and local hematoma at the block site.

## 3 Results

### 3.1 Perioperative analgesic efficacy

The infant's 24-hour postoperative FLACC pain scores were all in the mild pain range (≤2 points): 1 point at 1 h, 2 points at 4 h, 1 point at 6 h, 2 points at 12 h, and 1 point at 24 h. No FLACC score ≥4 points was recorded, and no rescue analgesics were administered.The first PCIA bolus was administered at 18 h postoperatively, with a total of 3 boluses within 24 h and a total tramadol consumption of only 70 mg (significantly lower than the 100∼200 mg tramadol consumption in traditional opioid-based analgesic regimens for pediatric DDH surgery reported in previous studies (1, 2). This prolonged time to the first rescue analgesia was consistent with the conclusion of a 2024 meta-analysis that Type Ⅱ QLB could significantly extend the interval of postoperative rescue analgesia in pediatric patients (3). The total intraoperative sufentanil dosage was 6 μg, achieving an opioid-sparing effect with satisfactory analgesia.

### 3.2 Perioperative vital signs and adverse reactions

Within 24 h postoperatively, the infant's vital signs remained stable with no obvious fluctuations: HR 95∼110 bpm, BP 80∼95/45∼60 mmHg, SpO₂ ≥ 98%. The infant woke up smoothly with no perioperative adverse reactions: no restlessness, nausea/vomiting, or urinary retention; left lower limb motor function was normal (independent flexion and extension, no motor block); no hematoma or infection at the lumbar block site. Parental satisfaction with perioperative anesthesia and analgesia was 9/10 (10-point scale).

### 3.3 Postoperative recovery outcomes

The infant achieved independent turning and sitting at 48 h postoperatively (early activity conducive to pediatric orthopedic recovery), with normal eating and defecation. No long-term complications (e.g., hip joint dysfunction, chronic pain) were observed during hospitalization. The infant was discharged uneventfully at 14 days postoperatively and had a good prognosis at follow-up.

## 4 Discussion

This case successfully applied a composite perioperative management strategy of general anesthesia combined with ultrasound-guided type Ⅱ QLB for a 19-month-old infant with DDH, achieving satisfactory analgesia, minimal opioid use, and no adverse reactions. The success of this strategy lies in the full consideration of the infant's specific anatomical and physiological characteristics and the optimization of perioperative management links for pediatric anesthesia challenges, which is highly consistent with the evidence-based practice of pediatric regional anesthesia and perioperative analgesia. We discuss the pediatric-specific perioperative management rationale, key operational points, clinical implications, and study limitations for DDH surgery combined with current evidence-based medicine below.

### 4.1 Rationale for ultrasound-guided type Ⅱ QLB in infant DDH surgery

Infant DDH surgery requires analgesic coverage of the hip joint, pelvic bone, and lateral thigh, and type Ⅱ QLB is the optimal regional block choice for this surgical area for three core reasons. First, wide analgesic coverage: Type Ⅱ QLB blocks the lumbar plexus (L1∼L4) and thoracolumbar sympathetic nerves, providing sensory analgesia for the hip, pelvic girdle, and lateral lower extremity, which is highly matched with the pain area of DDH open surgery (1). Second, pediatric safety advantages: Compared with traditional caudal block (20%∼30% urinary retention incidence in pediatrics), QLB has minimal impact on the sacral plexus, significantly reducing the risk of urinary retention (1); compared with fascia iliaca block, QLB provides more complete lumbar plexus coverage and longer-lasting analgesia (2). Third, ultrasound guidance for immature anatomy: A high-frequency linear probe clearly identifies the thin fascial layers and small muscles of infants, avoiding vascular/organ injury (e.g., abdominal cavity puncture) and ensuring accurate needle tip placement in the target space (4), which is the key to the safety of regional block in infants with underdeveloped anatomical structures.

The 0.2% ropivacaine dosage of 0.5 mL/kg used in this case strictly adheres to the *2025 Chinese Expert Consensus on Ultrasound-guided Nerve Block in Children* (recommended: ropivacaine 0.2%, 0.4∼0.6 mL/kg for pediatric QLB), which balances analgesic efficacy and the risk of local anesthetic toxicity—a critical concern in infants with immature liver metabolism. Slow injection and real-time ultrasound observation of drug diffusion further avoid local anesthetic accumulation and systemic toxicity.

### 4.2 Rationale for tramadol application and safety monitoring protocol

In this case, we used low-dose tramadol via PCIA for postoperative analgesia in a 19-month-old infant, and the selection of this regimen was based on sufficient evidence-based medical evidence and strict safety control. For opioid-sparing multimodal analgesia in pediatric orthopedic surgery, the 2023 WHO Guidelines for the Pharmacological Treatment of Persisting Pain in Children with Medical Illnesses and 2024 pediatric regional anesthesia consensus clearly state that low-dose tramadol via PCIA with strict dose control can be used in infants over 1 year old, after excluding contraindications and implementing standardized monitoring.

The infant in this case was 19 months old, with normal preoperative liver and kidney function, no history of abnormal drug metabolism, and ASA physical status Ⅰ, with no contraindications to tramadol. The tramadol regimen we used was an extremely low-dose design: background dose 0.17 mg·kg⁻^1^·h⁻^1^, single bolus 0.08 mg/kg, lock time 15 min, and the total daily dose was less than 2 mg/kg, which is far lower than the maximum recommended dose in international pediatric anesthesia guidelines. Compared with pure *μ*-opioid receptor agonists, tramadol has a dual analgesic mechanism, with a significantly lower risk of respiratory depression at equivalent analgesic doses, which is more suitable for infants with immature respiratory centers.

To ensure safety, we implemented a full-process standardized monitoring protocol: intraoperative continuous PETCO₂ monitoring; 30-minute continuous vital sign and sedation score monitoring in PACU after extubation; continuous ward monitoring of vital signs, respiratory rate and pattern within 24 h postoperatively, with FLACC pain score and Ramsay sedation score assessed every 4 h by trained nurses; and a pre-formulated emergency rescue protocol for respiratory depression. The infant had no adverse reactions such as respiratory depression, excessive sedation, or nausea/vomiting during the whole perioperative period, confirming the safety of this regimen.

### 4.3 Discussion on the prolonged analgesic duration

The first PCIA bolus was administered at 18 h postoperatively in this case, showing a prolonged effective analgesic duration, which is the result of the synergistic effect of the multimodal analgesia regimen with ultrasound-guided type Ⅱ QLB as the core. On the one hand, the single-shot type Ⅱ QLB with 0.2% ropivacaine provides the core foundation of long-acting analgesia. As a long-acting amide local anesthetic, 0.2% ropivacaine has a sensory block duration of 12∼24 h for single-shot pediatric peripheral nerve block, which is consistent with the 18-hour time point of the first analgesic requirement. This finding is strongly supported by a 2024 meta-analysis, which confirmed that Type Ⅱ QLB significantly extended the time to the first rescue analgesia in pediatric orthopedic surgery (3).On the other hand, the multimodal analgesia regimen further enhances and prolongs the analgesic effect through synergistic mechanisms: intraoperative low-dose sufentanil inhibits central sensitization caused by surgical stimulation; pre-emptive administration of low-dose tramadol PCIA before the end of surgery avoids the analgesic gap when the QLB effect declines; on-demand oral acetaminophen inhibits peripheral inflammatory pain caused by tissue injury. The combination of multiple analgesic mechanisms with different targets achieves a synergistic effect, further prolongs the analgesic duration, and reduces postoperative pain intensity.

### 4.4 Key points of perioperative management optimization for infant DDH surgery

Combined with this case and pediatric anesthesia guidelines, the core optimized measures for infant DDH surgery perioperative management are summarized as follows, which can provide a practical clinical reference for pediatric anesthetic practice: (1) Airway management: Use an uncuffed endotracheal tube for infants <2 years old, calculate intubation depth and ventilation parameters based on actual body weight, and continuously monitor PETCO₂ (the most important monitoring index for early detection of pediatric respiratory depression). (2) Regional block operation: For infant QLB, use a 6∼13 MHz high-frequency linear probe for superficial imaging; adopt in-plane puncture for real-time needle tip visualization; strictly control local anesthetic concentration/dosage and perform negative aspiration before injection. A 2024 systematic review of ultrasound-guided regional blocks in pediatric abdominal and hip surgery confirmed that standardized ultrasound operation could avoid severe complications in pediatric patients, and QLB was one of the most commonly used safe block techniques (7,8).(3) Multimodal analgesia: Combine ultrasound-guided QLB (regional analgesia) with low-dose PCIA (systemic analgesia) and on-demand oral acetaminophen to achieve an opioid-sparing effect, the first-line strategy for pediatric postoperative analgesia (4). (4) Pain assessment: Use the FLACC behavioral scale for non-verbal infants (the only validated objective pain evaluation tool) and set a clear rescue analgesia threshold (FLACC ≥4 points) to avoid undertreatment of pediatric pain. (5) Vital sign and adverse reaction monitoring: Maintain infant-specific vital sign ranges; focus on monitoring QLB-related complications (local hematoma, motor block) and opioid-related adverse reactions (respiratory depression, nausea/vomiting, urinary retention) within 24 h postoperatively.

### 4.5 Clinical warnings for pediatric QLB in DDH surgery

This case also provides important clinical warnings for the application of QLB in infant DDH surgery, which must be strictly followed to ensure safety: (1) Dosage precision is non-negotiable: Infants have a small body weight and immature liver metabolism; excessive local anesthetic can cause severe systemic toxicity (e.g., convulsions, cardiac arrest). Dosage must be calculated based on actual body weight, and the total dosage should not exceed the pediatric maximum recommended dose (ropivacaine ≤2 mg/kg). (2) Proficient pediatric anatomical knowledge: The infant lumbar fascial space is narrow and muscle boundaries are unclear; ultrasound operators must be proficient in pediatric-specific anatomical characteristics to avoid misplacing the needle tip into the abdominal cavity or blood vessels. (3) Avoid excessive sedation/analgesia: Infants have an immature respiratory center; the combination of general anesthetics and opioids may cause respiratory depression. Perioperative sedation and analgesia should be “adequate but not excessive”, with regular sedation score assessments. (4) Individualized PCIA setting: The PCIA background dose and single bolus for infants should be appropriately reduced to avoid drug accumulation caused by immature renal excretion function.

### 4.6 Study limitations

This study has the following limitations:

1. This is a single case report, and its conclusions lack verification from large-sample randomized controlled trials (RCTs), and the evidence level is limited; the relevant clinical efficacy conclusions cannot be directly generalized to all pediatric populations.
2. There is a potential measurement bias in this study. The FLACC pain scale, although a validated pediatric pain assessment tool, is a behavioral subjective assessment scale, which may have bias in the assessment process compared with objective pain monitoring indicators.
3. Ultrasound-guided QLB has obvious operator dependency. The success rate and efficacy of the block are closely related to the operator's proficiency in pediatric anatomical identification and ultrasound puncture technology, so the clinical application effect of this strategy may vary among operators with different experience levels.
4. In addition, the follow-up time is short, and the long-term effects of ultrasound-guided QLB in infant DDH surgery (e.g., chronic pain incidence, hip joint function recovery) require further long-term follow-up.

Future multi-center, large-sample RCTs are needed to compare the efficacy and safety of ultrasound-guided QLB with traditional analgesic regimens in pediatric DDH surgery, and to explore the optimal local anesthetic dosage and block type for different pediatric age groups. In addition, combined with the findings of recent meta-analyses and systematic reviews (3, 5), future studies should also focus on the long-term efficacy of Type Ⅱ QLB in reducing the incidence of chronic pain in pediatric DDH patients.

## 5 Conclusions

For the 19-month-old infant with DDH in this case, ultrasound-guided type Ⅱ QLB combined with general anesthesia is a safe and feasible perioperative management strategy, which can effectively cope with the pediatric-specific challenges of perioperative anesthesia and analgesia (anatomical immaturity, physiological sensitivity, pain assessment limitation). This strategy achieved satisfactory analgesic efficacy, a significant opioid-sparing effect, and no perioperative adverse reactions in this case, effectively improving the infant's postoperative comfort and parental satisfaction.

The key clinical experience for the successful application of this strategy in pediatric DDH surgery is summarized as follows: (1) Strictly follow pediatric anesthesia guidelines for precise drug dosage and airway management; (2) Use high-frequency ultrasound for real-time guidance to ensure accurate QLB puncture in infants with immature anatomy; (3) Adopt multimodal analgesia to balance analgesic efficacy and safety; (4) Conduct objective pain assessment with the FLACC scale and targeted monitoring of pediatric-specific adverse reactions.

Ultrasound-guided type Ⅱ QLB is a promising regional anesthesia technique for pediatric orthopedic anesthesia, and its standardized application in the perioperative management of infant DDH surgery has good clinical application prospects and is worthy of further clinical exploration and verification.For the special pediatric population, all perioperative management measures must be tailored to age, weight, and anatomical/physiological characteristics to achieve the goal of “safe anesthesia, effective analgesia, and rapid recovery”.

## Data Availability

The original contributions presented in the study are included in the article/Supplementary Material, further inquiries can be directed to the corresponding author.

## References

[1] Huang C Zhang X Dong C Lian C Li J Yu L. Postoperative analgesic effects of the quadratus lumborum block III and transversalis fascia plane block in paediatric patients with developmental dysplasia of the hip undergoing open reduction surgeries: a double-blinded randomised controlled trial. BMJ Open. (2021) 11(2):e038992. 10.1136/bmjopen-2020-038992 33542037 PMC7925863

[2] Yang YL. Effect of ultrasound-guided transmuscular quadratus lumborum block for postoperative analgesia in pediatric patient undergoing hip surgery. J Kunming Med Univ. (2023) 44(Suppl 2):1–6. 10.12259/j.issn.2095-610X.S20230426

[3] Park I Park JH Shin HJ Na HS Koo BW Ryu JH Postoperative analgesic effects of the quadratus lumborum block in pediatric patients: a systematic review and meta-analysis. Korean J Pediatr. (2024) 67(6):23268. 10.3344/kjp.23268 PMC10764215 38123185

[4] Chinese Expert Consensus Group on Ultrasound-guided Nerve Block in Children. Chinese Expert consensus on ultrasound-guided nerve block in children (2025 edition). Chin J Anesthesiol. (2025) 45(1):1–10.

[5] Zhu Y Wu J Qu S Jiang P Bohara C Li Y. Analgesic effects of quadratus lumborum block versus caudal block in pediatric patients undergoing abdominal surgery: a systematic review and meta-analysis. Front Pediatr. (2025) 13:1492876. 10.3389/fped.2025.1492876 39981211 PMC11839717

[6] Bauiomy H Kohaf NA Saad M Abosakaya AM. Comparison between peri-capsular nerve group and supra inguinal fascia iliaca block for analgesia and ease of positioning during neuraxial anesthesia in hip fracture patients: a randomized double-blind trial. Egypt J Anaesthesia. (2024) 40:193–200. 10.1080/11101849.2024.2333708

[7] Thanneeru SK Kiran M Padala SRAN Gupta A Ahmad R Chanchlani R Ultrasound-guided procedures for postoperative pain management in pediatric patients undergoing abdominal surgeries: a systematic review. Singapore J Anaesthesia. (2024) 28(3):951–60. 10.4103/sja.sja_951_23 PMC11033894 38654876

[8] Merkel SI Voepel-Lewis T Shayevitz JR Malviya S. The FLACC: a behavioral scale for scoring postoperative pain in young children. Pediatr Nurs. (1997) 23(3):293–7. 9220806

